# Supplementary material for: CIDER: Context-sensitive polarity measurement for short-form text
Source: PLoS One. 2024 Apr 18;19(4):e0299490. doi: 10.1371/journal.pone.0299490 (PMC11025856; doi:10.1371/journal.pone.0299490)
Supplement: S1 Appendix — (PDF) [file pone.0299490.s001.pdf]

Appendix

A Tweet Filters

Table 4. Filters used to separate general weather dataset into individual topics.

Dataset	Keep tweets containing	Exclude tweets containing	Tweet volume
**Heat**	‘sun’ OR ‘hot’ OR ‘heat’ OR ‘heat’ OR ‘summer’ OR ‘scorch’ OR ‘warm’ OR ‘fire’	‘cold’	7750
**Wind/ Rainfall**	‘wet’ OR ‘wind; OR ‘rain’ OR ‘storm’ OR ‘flood’ OR ‘water’ OR ‘hail’ OR ‘drizzle’ OR ‘tornado’ OR ‘hurricane’ OR ‘thunder’		8328
**Cold**	‘freez’ OR ‘frost’ OR ‘snow’ OR ‘cold’ OR ‘chilly’ OR ‘frozen’ OR ‘winter’ OR ‘blizzard’ OR ‘arctic’ OR ‘ice’ OR ‘icy’		2247

B Sentiment Classification

Table 5. Filters used to categorise individual sentiment models. These parameters have been taken from the respective documentations.

Method	Returns	Conversion
**CIDER**	float $\in$ (-1, 1)	Negative <-0.05, -0.05<Neutral<0.40, Positive >0.4
**Umigon**	‘positive’, ‘neutral’, ‘negative’	None
**Sentiment140**	0, 2, 4	0 = Negative, 2 = Neutral, 4 = Positive
**SentiStrength**	integer	Negative <0, Neutral = 0, Positive >0
**VADER**	decimal $\in$ (-1, 1)	Negative <-0.05, -0.05<Neutral<0.05, Positive >0.05
**TextBlob**	decimal $\in$ [-1,1]	Negative <0, Neutral = 0, Positive >0
**AFINN**	integer	Negative <0, Neutral = 0, Positive >0
**LIWC15**	posemo, negemo	posemo >negemo: positive, posemo <negemo: negative, posemo = negemo: neutral
**LIWC22**	tone_pos, tone_neg	tone_pos >tone_neg: positive, tone_pos <tone_neg: negative, tone_pos = tone_neg: neutral
