# Supplementary material for: CIDER: Context-sensitive polarity measurement for short-form text
Source: PLoS One. 2024 Apr 18;19(4):e0299490. doi: 10.1371/journal.pone.0299490 (PMC11025856; doi:10.1371/journal.pone.0299490)
Supplement: S1 File — (ZIP) [file pone.0299490.s002.zip › figures/PDF/VADERCIDER.pdf]

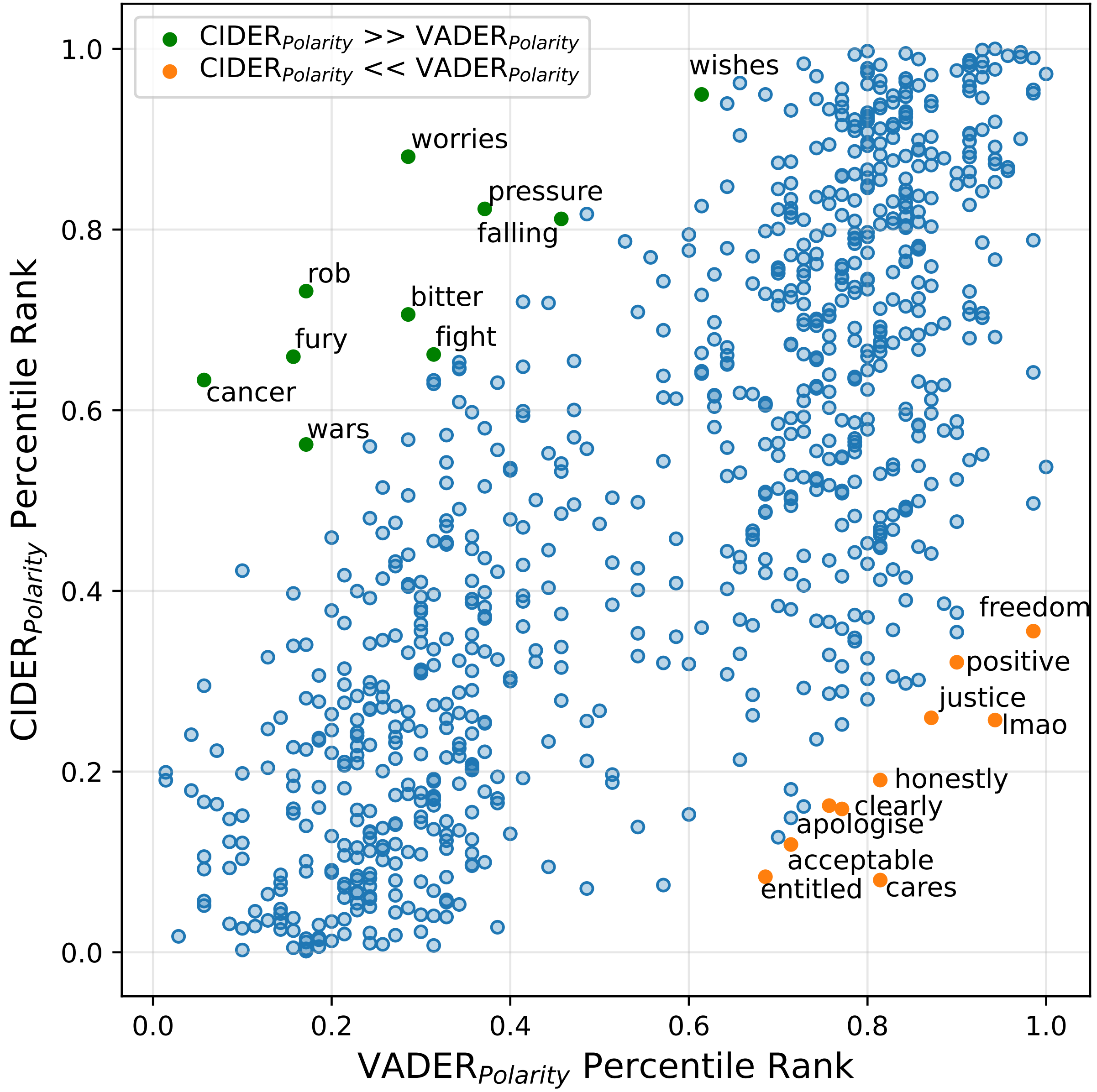

Word	Top 4 PPMI Words
**wars** ●	[star, maythe4thbewithyou, starwarsday, andor]
**fury** ●	[tyson, whyte, chisora, usyk]
**bitter** ●	[malty, bier, twisted, abv]
**worries** ●	[best, speedy, sending, belated]
**fight** ●	[billamsmithchamberlain, usyk, klub, fury]
**falling** ●	[mph, barometer, humidity, hpa]
**pressure** ●	[cumulus, 0mm, trend, °c]
**rob** ●	[rinder, burrow, lickey, enlarge]
**cancer** ●	[pancreatic, prostate, testicular, breast]
**worries** ●	[🐺, familyday, panics, mate]
...	...
**cares** ●	[nobody, noone, abortions, anymore]
**positive** ●	[tested, vibes, negative, affirmations]
**freedom** ●	[opiran, biafras, mahsaamini, iranians]
**honestly** ●	[foodbanks, defunding, 👁️, nevermind]
**clearly** ●	[concussed, onside, articulating, offside]
**justice** ●	[rajput, sushant, childabuse, mentoo]
**lmao** ●	[carats, lolz, wdym, wetin]
**entitled** ●	[opinion, £115k, brats, arrogant]
**acceptable** ●	[socially, perfectly, askingforafriend, deemed]
**apologise** ●	[profusely, inconvenience, molby, retract]
