# Supplementary material for: CIDER: Context-sensitive polarity measurement for short-form text
Source: PLoS One. 2024 Apr 18;19(4):e0299490. doi: 10.1371/journal.pone.0299490 (PMC11025856; doi:10.1371/journal.pone.0299490)
Supplement: S1 File — (ZIP) [file pone.0299490.s002.zip › figures/PDF/CIDER_PLOS_Submission_TABLES_1.pdf]

Text	CIDER Score	VADER Score
“Need AC - way too hot. Take care out there!!”	CIDER_heat: -0.652 (NEG)	VADER: 0.583 (POS)
“This storm is super scary. Please pray for us 🙏”	CIDER_wind: -0.633 (NEG)	VADER: 0.649 (POS)
“Drinking coffee watching the snow - It can’t get better than this!”	CIDER_cold: 0.438 (POS)	VADER: -0.402 (NEG)
