# Supplementary material for: CIDER: Context-sensitive polarity measurement for short-form text
Source: PLoS One. 2024 Apr 18;19(4):e0299490. doi: 10.1371/journal.pone.0299490 (PMC11025856; doi:10.1371/journal.pone.0299490)
Supplement: S1 File — (ZIP) [file pone.0299490.s002.zip › figures/PDF/CIDER_PLOS_Submission_TABLES_2.pdf]

**Text**	**Compound**	**Intensity**
Ronaldo is playing at Old Trafford today	-0.4251	0.5161
Out walking my 🐶 and a 🐱 ran towards us	0.2438	0.5168
Enjoyed watching the euros - was a great match 🦁	0.8139	0.8139
Wish this clown would accept retirement	-0.7804	0.7804
Support our school teachers and carers!	0.4217	0.4217
