# Supplementary figures and images for: CIDER: Context-sensitive polarity measurement for short-form text
Source: PLoS One. 2024 Apr 18;19(4):e0299490. doi: 10.1371/journal.pone.0299490 (PMC11025856; doi:10.1371/journal.pone.0299490)

# GeoUK 2022 Tweet Polarities

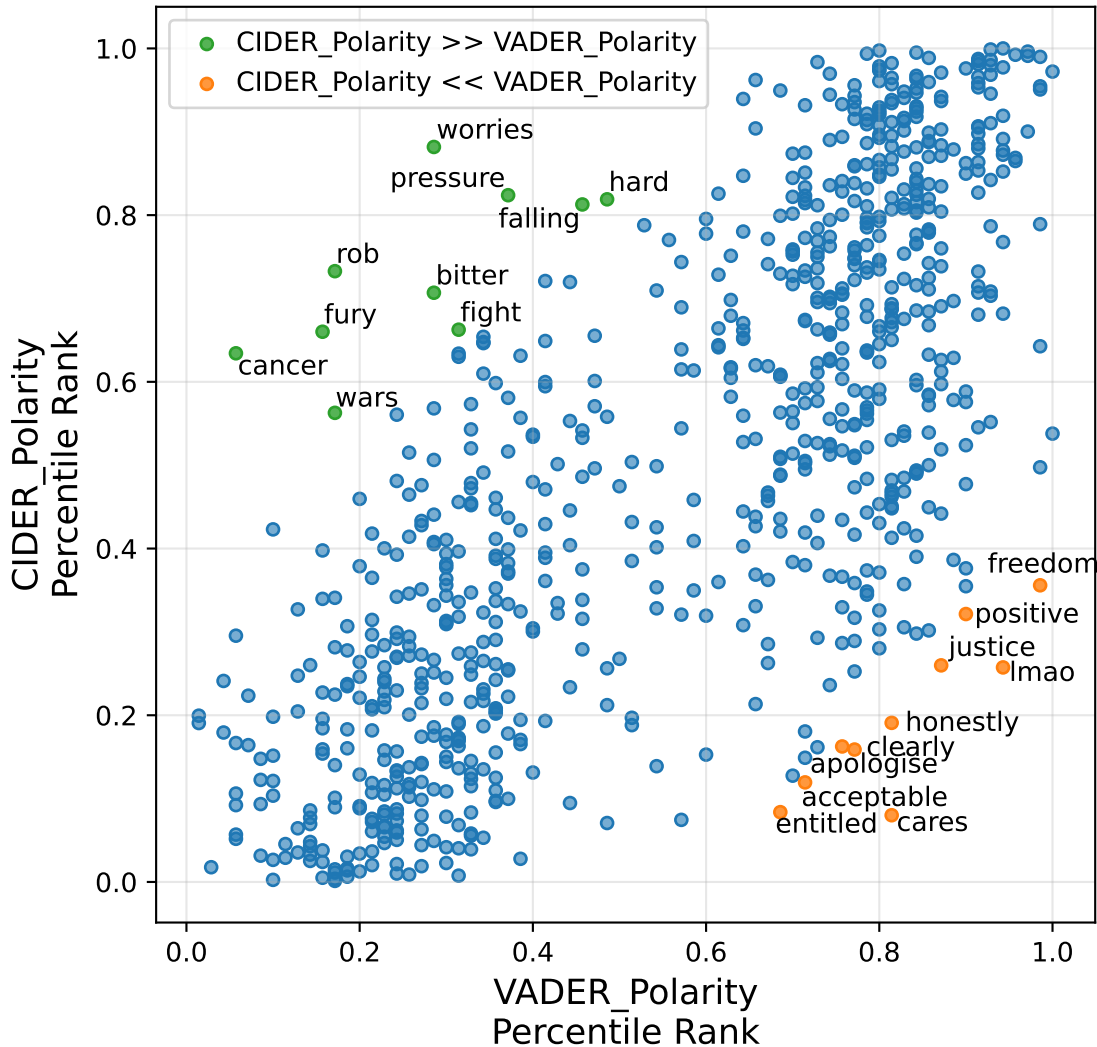

Supplement: S1 File — (ZIP) [file pone.0299490.s002.zip › figures/PDF/GeoUK_CIDER_VADER_compare.pdf]

Positive →

Sentiment

← Negative

4  
2  
0  
-2  
-4

Male ← Gender → Female

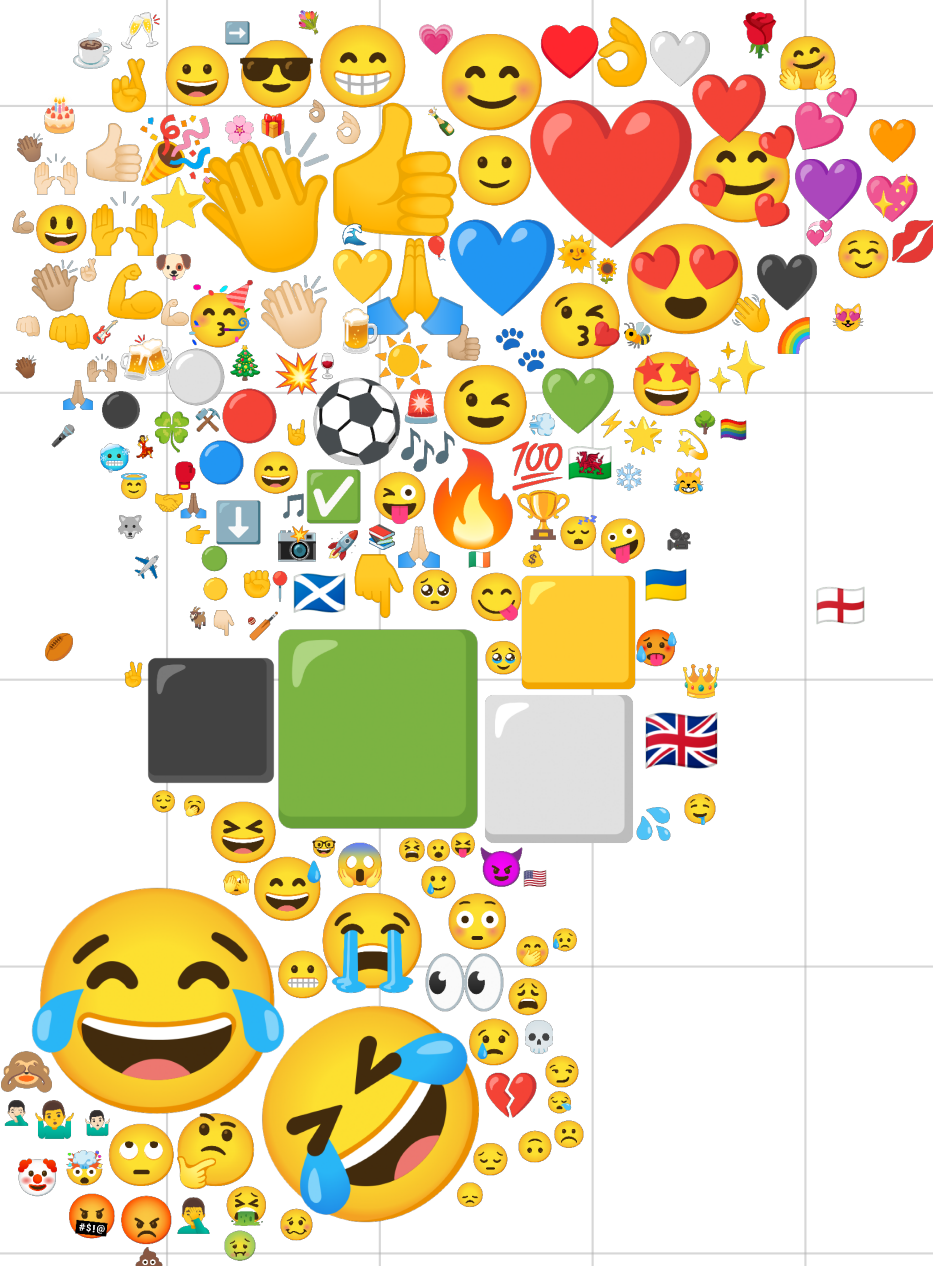

Supplement: S1 File — (ZIP) [file pone.0299490.s002.zip › figures/PDF/GeoUK_Emoji.pdf]

Daily Avg. Merged  
Intensity Detrended

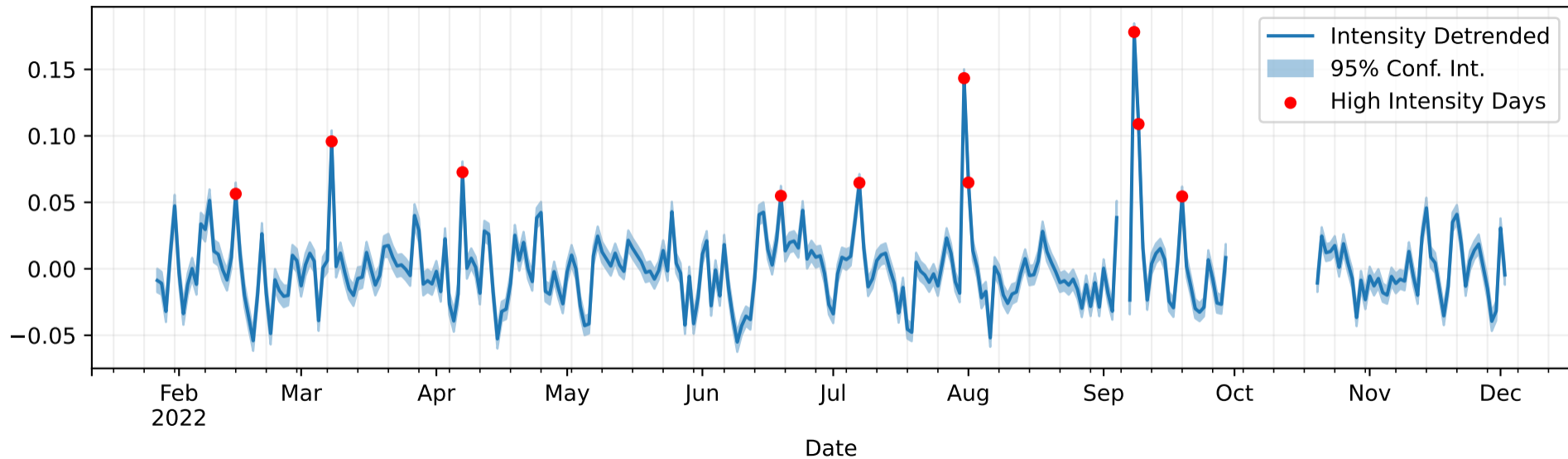

Supplement: S1 File — (ZIP) [file pone.0299490.s002.zip › figures/PDF/HighIntensityDays.pdf]

# Cold Weather Lexicon

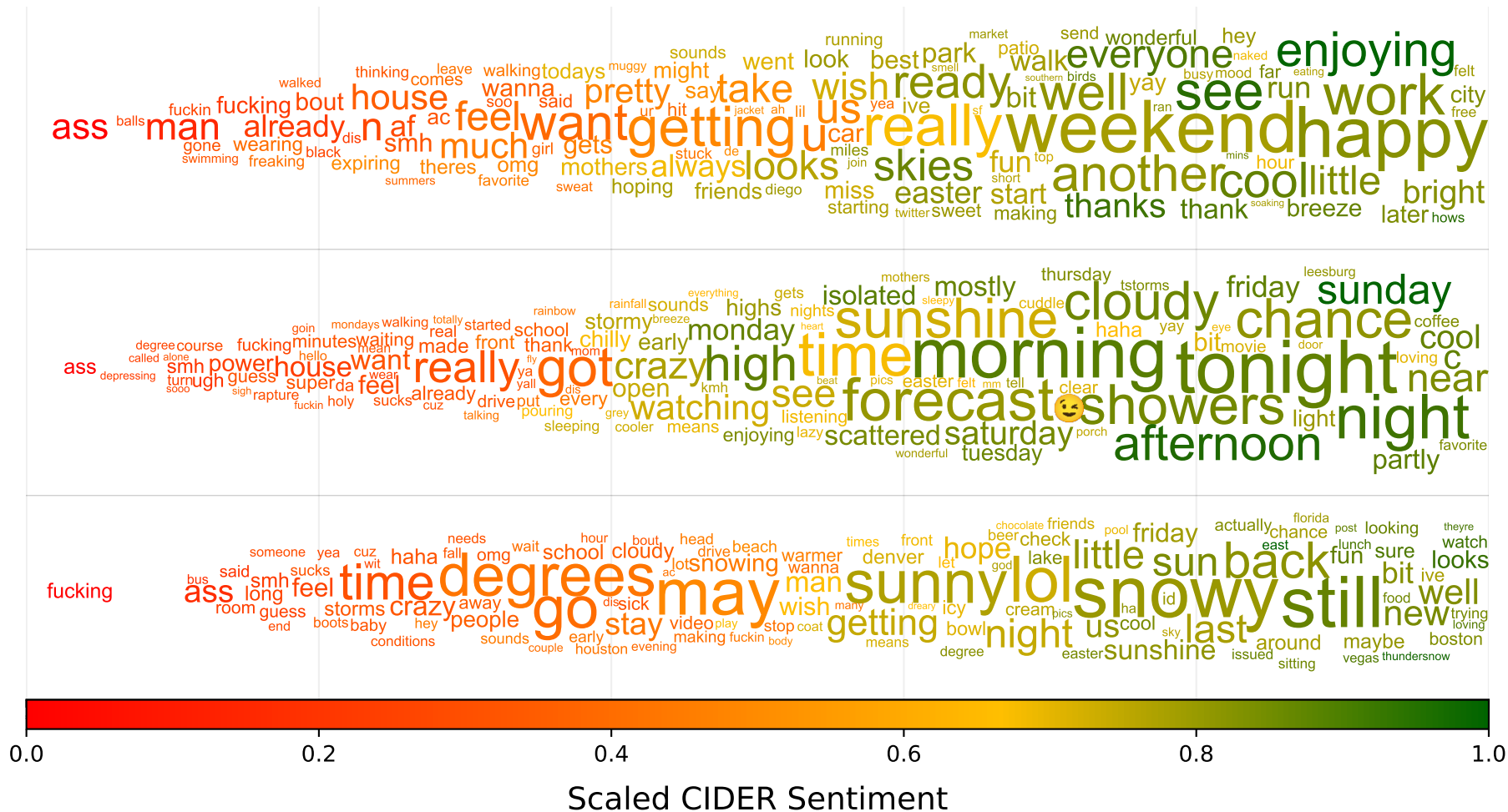

Supplement: S1 File — (ZIP) [file pone.0299490.s002.zip › figures/PDF/Weather_polarity_scales.pdf]
